# Ultrasounds and a Postharvest Photoperiod to Enhance the Synthesis of Sulforaphane and Antioxidants in Rocket Sprouts

**DOI:** 10.3390/antiox11081490

**Published:** 2022-07-29

**Authors:** Lorena Martínez-Zamora, Noelia Castillejo, Francisco Artés-Hernández

**Affiliations:** 1 Postharvest and Refrigeration Group, Department of Agronomical Engineering & Institute of Plant Biotechnology, Universidad Politécnica de Cartagena, 30203 Cartagena, Spain; lorena.martinez@upct.es (L.M.-Z.); noelia.castillejo@upct.es (N.C.); 2 Department of Food Technology, Nutrition, and Food Science, Faculty of Veterinary Sciences, University of Murcia, Espinardo, 30071 Murcia, Spain

**Keywords:** microgreens, germination, isothiocyanates, LED, illumination, light stress, shelf life

## Abstract

Ultrasounds (US) and LED illumination are being studied to optimize yield and quality. The objective was to evaluate the effect of a pre-sowing US treatment combined with a postharvest photoperiod including LEDs on rocket sprouts’ quality and phytochemicals during shelf life. A US treatment (35 kHz; 30 min) applied to seeds and a postharvest photoperiod of 14 h fluorescent light (FL) + 10 h White (W), Blue (B), Red (R) LEDs or Darkness (D) were assayed. Antioxidants as phenolics and sulfur compounds (glucosinolates and isothiocyanates) were periodically monitored over 14 days at 5 °C. The US treatment increased the sulforaphane content by ~4-fold compared to CTRL seeds and sprouts. The phenolic acids and the flavonoid biosynthesis were enhanced by ~25%, ~30%, and ~55% under photoperiods with W, B, and R, respectively, compared to darkness. The total glucosinolate content was increased by >25% (W) and >45% (B and R) compared to darkness, which also reported increases of ~2.7-fold (W), ~3.6-fold (B), and ~8-fold (R) of the sulforaphane content as a main isothiocyanate. Postharvest lighting is an interesting tool to stimulate the secondary metabolism, while a US treatment was able to increase the sulforaphane content in seeds and sprouts, although no synergistic effect was reported.

## 1. Introduction

Since the origins of humans, the development of agriculture has played a fundamental role in the development of humanity until our day, when the development of intensive and overgrown agriculture has contributed to a loss of diversity, desertification, and water consumption. For that reason, researcher groups around the world have developed techniques to reduce the environmental impact and to improve efficiency and agriculture yields, as one of the main Sustainable Development Goals -SDG- [1]. 

In this sense, the optimization of agriculture can positively affect poverty, hunger, diversity, climate change, unemployment, water uses, healthy living, security, women’s empowerment, desertification, energy, inequalities, consumption, protection, and the safeguarding of ecosystems, etc. Moreover, the research on new alternatives for veggie consumption is important for the need of promoting their health properties due to their high vitamin, minerals, antioxidants, and fiber content. In fact, the key function of antioxidant compounds in diet is to reduce the oxidative modifications of DNA, lipids, proteins, and carbohydrates as main mechanisms of cell damage, which can be the precursor of an illness [2]. In this sense, all these health-promoting compounds are related to the neutralization of free radicals and, hence, the reduction in the risk of suffering chronic diseases such as metabolic syndrome, inflammatory diseases, and even cancer [3].

In the middle of this scenario, the use of new eco-friendly technologies has demonstrated the optimization of agricultural production [4,5] while improving the secondary metabolism of plants, which is in charge of the production of phenolics, carotenoids, glucosinolates, and isothiocyanates.

To reach these goals, the development of new technologies has exponentially grown in recent years. Ultrasounds (US) have been incorporated into the industry to increase the extraction ability of some processes. Moreover, this technology acts by breaking the water molecules and making more available the bioactive compounds of the food matrix. Sonication is a way of generating energy by means of high-frequency sound waves (18 kHz–100 kHz) that cause damage and rupture in the membranes by cavitation [6]. When US was applied to soybean seeds, it was found that after germination, it increased the germination ratio, shoot length, and gamma-aminobutyric acid content [7], showing possible effects that this technology can induce in sprouts, even before sowing.

Light is essential to the development of the plant, but the characteristics (quality and quantity) of the applied light during growing, and during the postharvest period, can afford a better bioactive quality for the horticultural commodities. In this sense, the application of specific wavelengths of the visible spectrum with Light Emitting Diodes (LEDs) has been effective during the germination of plants in their earlier stages, such as sprouts [8,9], and also during a refrigerated shelf life period [10,11]. Moreover, during cold storage and retail sale periods, shorter (from 400 to 500 nm) and longer (from 600 to 700 nm) wavelengths of the visible light spectrum have shown to be effective in the bio-production of health-promoting compounds in adult fruit and vegetables [12,13,14].

Such technologies could be incorporated to the development of young plants, whose bioactive compounds can reach up to 10-fold the ones in the adult plant. Regarding sprouts and microgreens, *Brassicaceae* sprouts are considered one of the healthiest species due to their richness in isothiocyanates with important anti-inflammatory and anticancer properties [15,16]. Particularly, rocket sprouts are not still well known by consumers, who are used to consuming their baby leaves, but their richness in flavonoids and glucosinolates makes them an interesting commodity with excellent health benefits.

Therefore, the main objective of this work was to evaluate the effect of the application of a US treatment to the seeds before sowing, combined with a postharvest photoperiod with several LEDs, on rocket sprouts’ quality and their main bioactive compounds changes during a refrigerated shelf life period.

## 2. Material and Methods

### 2.1. Plant Material

Rocket (*Eruca sativa*) seeds were supplied by Intersemillas S.A. (Valencia, Spain). Three grams of rocket seeds were weighed and washed with 40 mL of autoclaved distilled water for 2 h.

### 2.2. Ultrasound Seed Treatment

Rocket seeds in water were treated for 30 min at room temperature with 35 kHz ultrasound (US) (Bandelin Sonorex Digiplus DL 514 BH, Berlin, Germany). Seeds were divided into two treatments: US treated and not treated, which were used as control (CTRL).

### 2.3. Seed Germination and Minimal Processing 

A laminar flow cabinet (Telstar Bio-II-A/M, Japan) was used for sowing, where both seed treatments were arranged in polypropylene trays (TR-370; 118 × 96 × 39 mm). Subsequently, we used a filter paper as a support at the bottom of the tray, which were moistened with autoclaved distilled water, and a 40 μm film partially covered the tray to ensure high relative humidity (RH) in the trays. These trays were placed prior to seeding on a UV-C light table for 30 min to be sterilized. Conditioned trays with sprouts were kept in the germination chamber (Sanyo MLR-350 H, Japan) for 7 days at 20 °C, 90% HR, and under darkness conditions. During this period, sprouts were irrigated twice per day.

On the 7th growing day, sprouts were minimally processed in a cold room at 12 °C, wherein they were disinfected for 1 min with a 150 ppm sodium hypochlorite solution and rinsed in cold water for 1 min. They were then placed on absorbent filter paper to promote drying before be packaged in trays (TR-250; 101 × 81 × 35 mm) sealed with 30 μm bi-Oriented Polypropylene (OPP) film under passive modified atmosphere packaging (MAP). OPP film and trays were previously cooled for 24 h to avoid condensation. The permeability of the OPP film was 1100 cm^3^ m^−2^ d^−1^ atm^−1^ (O_2_) (23 °C, 0% RH; data provided by the supplier according to DIN 53380) with a coefficient of permeability equal to 1.

On harvesting day, 50 sprouts were characterized, and one replicate consisted of 10 sprouts. The sprouts were arranged horizontally near a ruler and photographed. The photographs were processed using Image J software (Wayne Resband, MD, USA) to obtain the length of the hypocotyl (H) and root (R) of the sprouts. The results were presented in cm. The growth rate was calculated by dividing H (mm) by the days of growth (d), expressing the results in mm/d.

### 2.4. Postharvest Lighting Treatments

After packaging, the trays with the sprouts were stored for 14 days at 5 °C under illumination conditions of 14 h fluorescent light (FL) in all cases + 10 h LED photoperiod, simulating a retail sale period in supermarkets where the lights are usually turned off during the nights for a period of around 10 h. The FL light intensity applied was 7 W m^−2^ for 14 h per day (daily dose of 352.8 kJ m^−2^). LED lights used in the photoperiod were switched on for 10 h with an intensity of 10.35 W m^−2^ (daily dose of 372.6 kJ m^−2^). Therefore, the postharvest photoperiod lighting treatments were: FL + Darkness (D), FL + White (W), FL + Blue (B), and FL + Red (R). Sampling days were on 0, 5, 8, and 14 days at 5 °C. The light spectra applied are specifically detailed in Figure S1. Two experiments were carried out simultaneously, one with the sprouts obtained from US-treated seeds, and the other one with the sprouts from non-treated US seeds. Twelve trays of 10 g of sprouts were stored under each light treatment. Each tray represented a replicate, and four replicates were taken for each sampling time.

### 2.5. Physiological Quality during Shelf-Life 

Weight losses were determined based on the weight of the sprouts at harvest. All trays were weighed on the initial day and on each of the sampling days (day 5, 8, and 14). Four trays were weighed per treatment and sampling day. Results were expressed as %.

The O_2_ and CO_2_ partial pressures within trays were periodically monitored during shelf life. Samples of 1 mL were taken with a gas-tight syringe from the headspace and injected into a gas chromatograph (Precisely Clarus500, PerkinElmer). On each analysis day, a standard with a known composition of 8.04 kPa CO_2_ and 10.03 kPa O_2_ was used in the identification and quantification of the O_2_ and CO_2_ peaks. Four trays were analyzed per treatment and sampling day.

### 2.6. Bioactive Compounds Determination

#### 2.6.1. Antioxidant Compounds: Phenolic Acids and Flavonoids

A total of 3 mL of methanol:water (80:20, *v*/*v*) was added to 25 mg of freeze-dried rocket sprouts. These samples were shaken for 1 h on an orbital shaker (Stuart, Stone, UK) inside a polystyrene box with a bottom of ice and covered with aluminium foil to protect them from light. Samples were centrifuged at 3220× *g* for 10 min at 5 °C. The supernatant was collected, and 1 mL of the methanolic extract was filtered using 0.2 µm polytetrafluoroethylene membrane filters. An Ultra High Performance Liquid Chromatography (UHPLC) instrument (Shimadzu, Kyoto, Japan) equipped and conditioned as described by Castillejo et al. [17] was used. Each sample was analyzed in triplicate (N = 3). The absorption spectra were recorded between 200 nm and 400 nm, and the results were expressed as g kg^−1^ by using analytical standards supplied by Sigma. The total phenolic acid content was calculated by the sum of Gallic acid, Caffeic acid, Ferulic acid, Coumaric acid, and Sinapic acid. The total flavonoid content was calculated by the sum of Kaempferol derivatives (1, 2, and 3), Rutin, Quercetin, and Quercetin derivative.

#### 2.6.2. Glucosinolates

Extraction, analysis, identification, and quantification of desulfoglucosinolates were carried out following the method described by Martínez-Zamora et al. [18]. For the identification and quantification, a UHPLC instrument (Shimadzu, Kyoto, Japan) equipped as described by Martínez-Zamora et al. [18] was used. A volume of 20 µL of the extracted samples was injected into the system, where water was the phase A and acetonitrile the phase B. The flow rate was 1.5 mL min^−1^, and the gradient followed for phase A was 0:100, 28:80, 30:100 (min/% A). Desulfoglucosinolates were detected at 227 nm, and the results were expressed in g kg^−1^ as the mean of three replicates.

#### 2.6.3. Sulforaphane

Sulforaphane was extracted and analyzed following the method described by Martínez-Zamora et al. [18]. For that, 5 μL sulforaphane extracts were injected in a UHPLC (Shimadzu, Kyoto, Japan) equipped as described above in the glucosinolate methodology with a Gemini C18 column (250 mm × 4.6 mm, 5 µm particle size; Phenomenex, Torrance, CA, USA). The mobile phases, 0.02 mol L^−1^ ammonium formate (A) and acetonitrile (B) with a 0.6 mL min^−1^ flow rate, were prepared to detect sulforaphane at 196 nm. DL-sulforaphane provided by Sigma-Aldrich (St. Louis, MO, USA) was used as a standard, and results expressed as g kg^−1^ were obtained as the mean of three replicates.

### 2.7. Statistical Analysis

The experimental design was a three-factor analysis (US treatment T, photoperiod P, and storage time *t*) where analysis of variance (ANOVA) was performed using Statgraphics Plus software (vs. 5.1, Statpoint Technologies Inc., Warrenton, VA, USA). Statistical significance was set at *p* ≤ 0.05, and Tukey’s multiple range test was used to establish and separate means.

## 3. Results and Discussion

### 3.1. US Seed Treatment Effect

One hour after the US treatment was applied to rocket seeds before sowing, the main bioactive compounds of US-treated and CTRL seeds were analyzed, with results shown in Table 1.

The total phenolic acid content in the CTRL rocket seeds was 13.23 ± 0.60 mg g^−1^, from which the major compound was gallic acid (~27%) followed by coumaric acid (~23%), sinapic acid (~22.4%), ferulic acid (~13.8%), and caffeic acid (~13.8%). The total flavonoid content was 35.15 ± 1.50 mg g^−1^, from which the major compound was quercetin (~32.2%) followed by quercetin derivative (~26.4%), kaempferol derivative-2 (~17.5%), kaempferol derivative-3 (~11.8%), rutin (~6.4%), and kaempferol derivative-1 (~5.7%). 

In those cases, US treatment tended to reduce the concentration of phenolic compounds in treated seeds compared to the CTRL. A reduction of almost ~28% of the phenolic acids and ~49.4% of flavonoids was found in such seeds after US treatment, which indicates that this technology, applied at the described conditions, did not report an improvement in the biosynthesis of phenolic compounds, and it should be further investigated to test its benefits. As a matter of fact, the extraction parameters such as temperature, time, frequency, power, and type of solvent are essential factors to control in the extraction of bioactive compounds by applying US [19], and the origin or the cultivar also affect the phytochemical profile. In this sense, although US technology helps to recover bioactive compounds from a different food matrix, it is necessary to also control parameters such as color, pH, or particle size of suspension, which also influence the breaking of the chemical bond of bioactive compounds. For that reason, we can conclude that the studied US conditions in the present study are not adequate to extracts flavonoids, although they were more effective in extracting sulforaphane, the most interesting compound in the studied seeds.

The major compounds in these *Brassicaceae* seeds were glucosinolates and sulforaphane as the main isothiocyanate, whose biosynthesis was indeed positively affected by the application of US. In this case, the total glucosinolate content of CTRL seeds was 35.6 ± 1.44 mg g^−1^, from which the major compound was dsf-glucoerucin with ~66.5%, followed by dsf-glucoraphanin with ~33.5%. One hour after the US treatment, the treated seeds saw an increase in the total glucosinolate content by ~10%. Although this increase was not significant, this tendency was more remarkable after the derivation of glucoraphanin into sulforaphane. In fact, it seems that the 35 kHz US bath for 30 min stimulated the action of the myrosinase enzyme to make the conversion into this bioactive molecule, because the biosynthesized sulforaphane content was 4.4-fold higher in US rocket seeds compared to the CTRL.

In this sense, recent research has shown that the application of US can shorten and improve the extraction rate of sulforaphane from broccoli seeds [20]. This behavior was attributed by the authors to the high efficiency of enzymolysis processes and solubilization, which is induced by the mechanical and cavitation effects produced by this technology. In fact, in this study [20], the extraction rate of sulforaphane was increased by ~4-fold by applying US, which demonstrates the potential application of US to sulforaphane extraction in *Brassicaceae* seeds.

### 3.2. Physiological Changes during Shelf Life

**Figure 1:**
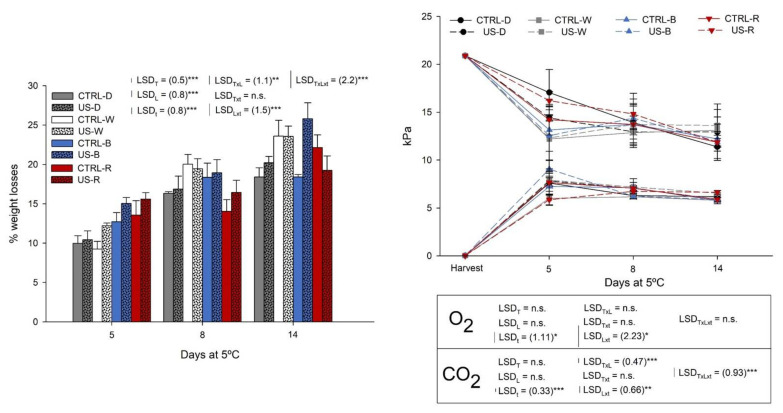
Weight losses (**left**) and gas partial pressures within trays (**right**) of rocket sprouts from control (CTRL) and ultrasound treated seeds (US) for 14 days at 5 °C under illumination with a photoperiod of 14 h fluorescent light (FL) + 10 h with White (W), Blue (B), Red (R) LEDs or in Darkness (D). T: seed treatment; L: light treatment; t: sampling time; *: *p* < 0.05; **: *p* < 0.005; ***: *p* < 0.001; ns: not significant.

The physiological changes of rocket sprouts under different LED photoperiods during the refrigerated shelf life were evaluated by the determination of dehydration and the atmospheric composition within packages, whose results are shown in Figure 1.

The weight losses of rocket sprouts stored for 5 days at 5 °C in darkness conditions were 10%, without differences among the US-treated or CTRL seeds (Figure 1-left). On the same day, the sprouts stored under different light photoperiods reported higher weight losses compared to the CTRL. In this sense, B and R lights induced a ~13 and ~14% weight loss in the rocket sprouts. The US treatment on rocket seeds did not report an important effect on subsequent sprout weight losses, and the interaction of this treatment with days of shelf life was not significant. However, the interaction of the three studied factors (seed treatment, postharvest light, and time) was significant. Generally, a great effect of the US treatment on the weight loss during the shelf life of rocket sprouts cannot be found.

Weight losses were increased during shelf life, reporting higher dehydration, and leading to senescence. After 8 days, the lowest weight losses were monitored in sprouts stored in darkness conditions, with ~16%, and increased to ~18% after 14 days at 5 °C. Regarding all studied photoperiods, W and B LEDs, especially, reported higher water losses than those sprouts under darkness conditions. This behavior has been previously described and can be due to the fact that the plant metabolism is continuously activated under light conditions [10,21,22].

As appreciated in Figure 1-right, the atmospheric composition within MAP samples followed the expected tendency. Oxygen partial pressures decreased from 21 kPa to 12–14 kPa, without relevant differences among illumination treatments. CO_2_ partial pressures stabilized at 7–8 kPa, with no differences between the studied photoperiods. However, a slight tendency could be distinguished after 5 days, when the O_2_ consumed by rocket sprouts under R and D photoperiods was slightly lower than that under B and W illumination. This behavior may be shown by the fact that wider wavelengths (R) could reduce the respiration rate of sprouts at the beginning of the storage, when breath gases are still not stabilized. Similar results were recently found by Zhang et al. [23] when storing pakchoi for 30 days under 30 μmoL m^−2^ s^−1^ red + white LEDs, combined with different modified atmosphere packages (MAP): 5 kPa O_2_ + 10 kPa CO_2_ + 85 kPa N_2_; 5 kPa O_2_ + 5 kPa CO_2_ + 90 kPa N_2_; and 10 kPa O_2_ + 5 kPa CO_2_ + 85 kPa N_2_. Nevertheless, as no significant differences were found among lighting treatments in the present study, no conclusions can be reached when the normal partial pressures of these gases are reached in the steady state after 8 days at 5 °C, with the consequent chloroplasts’ degradation, the reduction in inorganic salts and enzyme activities, which leads to the decline in the quality of vegetables, the stopping of the photosynthesis, and hence the decrease in the respiration rate [23,24].

### 3.3. Bioactive Compound Changes during Shelf Life under Illumination Treatments

#### 3.3.1. Phenolic Compounds

Regarding the biosynthesis of phenolic compounds during the refrigerated storage of rocket sprouts, Table 2 and Table 3 show the evolution of phenolic acid compounds and flavonoids, respectively, while the statistic for simple factors and their interactions is presented in Table 4. When comparing the obtained values on rocket seeds, we can observe that the phenolic compound biosynthesis has been reduced 10-fold due to the reduction in the dry matter of the sprouts, which increased their water content during germination. Similarly, as described in Table 1, no differences were found between sprouts from US-treated and CTRL seeds, which support our previous theory that the US technology applied at these conditions did not affect the biosynthesis of phenolic acids, either on the seeds or their sprouts. By contrast, the different photoperiods assayed throughout the shelf life were able to increase the biosynthesis of both phenolic compounds: phenolic acids and flavonoids. Polyphenols are involved in the defense system of plants and protect them from photooxidation [25]. That is, the total phenolic acid content was increased during the postharvest period (for 14 days at 5 °C) in CTRL rocket sprouts by ~22% under W, ~27% under B, and ~43% under R compared to darkness conditions. A similar effect was found in US-treated sprouts, which increased their phenolic acid content by ~21% under W, ~28% under B, and ~23% under R compared to darkness.

Specifically, five different phenolic acids have been identified. From a higher to lower amount, they are coumaric acid, ferulic acid, caffeic acid, sinapic acid, and gallic acid (Table 2). As observed, no significant differences were found between different light photoperiods and US-seed treatments after 5 days at 5 °C in any phenolic acids identified. After that, increases by ~30% for the coumaric acid were found under W and R photoperiods and by ~40% under B compared to darkness. Higher differences were appreciated regarding the content of ferulic acid, which increased to twice its quantity under R, and by ~62% under B, both compared to darkness conditions. Moreover, the production of this compound was ~36–45% higher under a W than under a D photoperiod. With regards to caffeic acid content, it was also stimulated by ~50%, ~60%, and ~93% under W, B, and R, respectively, compared to D. Nevertheless, the production of sinapic acid and gallic acid was less stimulated, because only R light during postharvest improved by ~24% the content of gallic acid compared to darkness conditions.

Other authors have obtained similar results regarding phenols accumulation after 5 days of broccoli germination under red and blue LED light treatments applied for 24 h [26]. It can be presumed that the metabolic pathway of broccoli phenols varies according to shoot growth, and that it can reach a higher accumulation under yellow LED light after 15 days at 5 °C, as recently reported by Castillejo et al. [11]. However, in soybean, it has been shown that the total phenol content increases under green and blue LED lights, with blue lights reported to have the higher content [27]. Therefore, the increase in phenols varies according to the treated species and the relationship between cryptochromes and phytochromes with the synthesis of such compounds. In the case of rocket, it was increased in all studied photoperiods, especially when FL was complemented during the night with blue and red LEDs.

According to the secondary metabolism pathway, we can observe as the illumination partially affects the beginning of the chain (gallic acid production and its derivatives), but it notably improves the synthesis of other compounds produced in the middle of the chain and cofactors that are the predecessor of the flavonoid production, with interesting results due to the potential role of phenolic compounds in the antioxidant activity of the diet [2]. This fact reflects that the light presence during the postharvest period (transportation and retail sale in supermarkets) can preserve the number of phenolic acids, which can be observed according to data reported by our darkness treatment, both for CTRL and US. Therefore, if we do not switch off the lights and we keep them on during the night periods, this amount can be even doubled. All lighting treatments studied (W, B, and R), reported an increase in the biosynthesis of these compounds during the shelf life, although it is important to remark that the extreme wavelengths of the visible spectrum (B and R) individually applied showed better results than the full spectrum (W). This behavior corroborates what has been recently reported in broccoli sprouts [10,11], which also belongs to the *Brassicaceae* family, or in carrot sprouts [18].

Regarding the major phenolic compounds found in *Eruca sativa*, it is remarkable that no positive effects were found until 8 days at 5 °C in the flavonoid biosynthesis during shelf life, because the induced abiotic stress is still incipient. Nevertheless, we can appreciate how after said days, the biosynthesis of these antioxidant compounds in CTRL rocket sprouts was increased by ~21%, ~19%, and ~57% under W, B, and R light photoperiods compared to D, respectively. Moreover, in US-treated sprouts, this increase is also appreciated, although in a lower amount. In this case, rocket sprouts under W reported an increase of ~10% in their flavonoid content, while B and R did it by ~20% and ~22%, respectively, in comparison with darkness. As observed, such increases are lower due to the improvement of the flavonoid content in US-D sprouts compared CTRL-D. It seems that the remanent effect of the US treatment on rocket seeds may be effective to increase the content of these compounds, which was translated as an enhancement by ~23% compared to those sprouts from US-untreated seeds. Therefore, we cannot close the door to the possibility of a useful application of US as a pre-treatment for seeds before sowing, which should be further studied.

Six different flavonoids with high antioxidant ability have been identified in rocket sprouts, from which quercetin reported the highest content followed by kaempferol derivatives (1,2,3, all of them attached to mono- and disaccharide sugars), rutin, and a quercetin derivative (attached to mono- or disaccharide sugars), while kaempferol was found in very low amounts (Table 3), which agrees with those reported by Cuellar et al. [28] and Schiavon et al. [29], who found such compounds in similar contents in rocket leaves. Similar to phenolic acids, flavonoid synthesis was not enhanced at the beginning of the shelf life (5th day), although the stimuli of these compounds induced by lighting conditions was notorious after the 8^th^ day. In this sense, W, B, and R improved the synthesis of quercetin and kaempferol derivative-2 by ~23%, ~18%, and ~35%, respectively, compared to darkness, while lower increases affected the kaempferol derivatives (1,3) and quercetin derivative, whose production was increased by ~17%, ~10%, and ~29% under W, B, and R, respectively, compared to darkness. Lastly, the most positively affected flavonoid by the postharvest illumination was rutin, which increased its content by ~27% under W, ~33% under B, and ~55% under R in comparison with darkness. This behavior can be explained by the fact that plant photoreceptors absorb blue (cryptochromes) and red lights (phytochromes and phototropins), which activate the gene transcription factors involved in the photomorphogenesis of phenolic and flavonoids [30]. These mechanisms are triggered by the PSY genetic chain, which regulates the overexpression of HY5, on whose work depends the synthesis of phytochemical compounds with high antioxidant capacity from the plant’s secondary metabolism [31,32,33]. Therefore, the supplementation of the normal photoperiod with W, B, and R light can also improve the concentration of these compounds, which demonstrates the positive effect of the continuous illumination on maintaining as active the secondary metabolism of the plant, even after it harvest.

Therefore, it is possible to conclude that as the main responsibility for the antioxidant activity of fruits and vegetables [2,34], the enhancement of the biosynthesis of phenolic acids and flavonoids by the use of LED lighting during the storage of rocket sprouts is going to suppose the improvement of the healthiness of these little plants, being more able to scavenge free radicals present in diets and avoiding the development of possible new diseases produced by oxidative stress to which the human body is subjected on a daily routine [2,3].

Nevertheless, as previously cited, these increases in the flavonoid biosynthesis of rocket sprouts from US-treated seeds was indeed positively affected by such technology by ~20%, which was also observed in the concentration of the individual flavonoids of CTRL-D and US-D, possibly due to its remanent effect. Nevertheless, although these results are promising, this novel technology as a pre-treatment for the seed before sowing must be still studied to reach strong conclusions regarding its use.

#### 3.3.2. Glucosinolates

**Figure 2:**
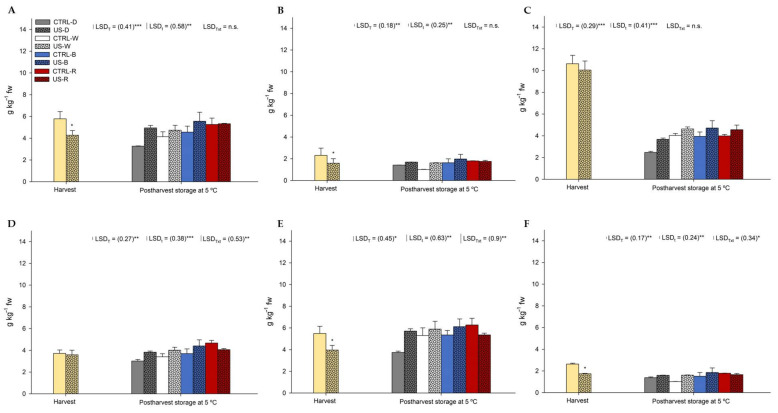
Dsf-glucoraphanin (**A**), Sinigrin (**B**), Dsf-Glucoerucin (**C**), Dsf-4-methoxy-glucobrassicin (**D**), Dsf-Glucobrassicin (**E**), Dsf-Neoglucobrassicin (**F**) content of rocket sprouts obtained from untreated (CTRL) and treated seeds (US) during a shelf life of 14 days at 5 °C (mean of obtained values from each sampling time) under illumination with a photoperiod of 14 h fluorescent light + 10 h with several visible spectrum LEDs or in darkness. *: *p* < 0.05; **: *p* < 0.005; ***: *p* < 0.001; ns: not significant.

The main glucosinolates found in rocket sprouts by elution order were glucoraphanin (Figure 2A), sinigrin (Figure 2B), glucoerucin (Figure 2C), 4-methoxy-glucobrassicin (Figure 2D), glucobrassicin (Figure 2E), and neoglucobrassicin (Figure 2F). Obtained results on sampling days during shelf life did not show significant differences, for which reason a mean of the obtained values is shown in Figure 2. As the sum of all the identified compounds, the total glucosinolate content of CTRL rocket sprouts at harvest was 30.55 ± 2.47 g kg^−1^, while the US was 25.20 ± 2.15 g kg^−1^, which again showed that a US treatment of seeds did not report any effect on the glucosinolates biosynthesis. The total glucosinolate content of rocket sprouts decreased by ~50% compared to the initial content in those samples stored under the darkness photoperiod. By contrast, the incorporation of B and R lights into the photoperiod assayed maintained the glucosinolate level at a similar content than that registered at harvest. 

According to data shown in Figure 2, dsf-glucobrassicin content represented 25% of the total glucosinolate content, followed by dsf-glucoraphanin with 22%, dsf-glucoerucin and dsf-4-methoxy-glucobrassicin with 18.5% each, and sinigrin and dsf-neoglucobrassicin with 8% each. In this sense, all the glucosinolate compounds followed the same behavior. Although a US pre-treatment to the seeds did not increase the glucosinolate content, the postharvest LED illumination photoperiod did compared to darkness. CTRL rocket sprouts showed an increase by >25% when W lights were used in the photoperiod, while B and R lighting reported increases by >45% compared to darkness. The individual glucosinolates mostly affected by LED lighting were glucoraphanin, glucoerucin, glucobrassicin, and 4-methoxy-glucobrassicin, whose sum represented 84% of the total glucosinolate content. Moreover, neoglucobrassicin and sinigrin were positively affected when W (~10%), B (~25%), and R (~25%) LEDs were included in the photoperiod applied during the shelf life. 

Regarding the stimuli of glucosinolate production, short wavelengths near blue have demonstrated to be useful to increase the biosynthesis of these compounds, since UV-B and blue lighting are good elicitors of this secondary metabolite production [18,35,36]. Furthermore, Wang et al. [37] have recently shown that 4 weeks of LED illumination (with red and red/blue lighting) can stimulate the glucosinolate and sulforaphane biosynthesis in broccoli seedlings. Obtained results by cited authors showed that red light induced the expression of cofactor genes involved in the biosynthesis of aliphatic and indole glucosinolates. In that case, the illumination under red and the combined red and blue LEDs during the growth of broccoli seedlings reported an upregulation of CYP79B2, CYP79B3, and CYP83B1 summed to the hyperproduction of the tryptophan, from which indole forms are derived from. In addition, the hyperexpression of SOT18 induced the production of the aliphatic forms of these compounds [37]. Specifically, five genes (MAM1, CYP83A1, UGT74C1, SOT17, and SOT18) were upregulated under red and blue LED lighting [37], which could explain the obtained results in the present study regarding the increased content of aliphatic glucosinolates (Figure 2A–C; sinigrin, glucoraphanin, and glucoerucin) under red lighting. Apart from that, many studies have shown that once the regulator of the secondary metabolism is HY5 is activated by photoreceptors, its overexpression can increase the formation of phenolic compounds, carotenoids, chlorophylls, anthocyanins, and glucosinolates [38,39].

The most interesting fact of glucosinolates is that isothiocyanates are derived from them, which are powerful against inflammation and human cancer development. Specifically, sulforaphane is an isothiocyanate derived from the conversion of glucoraphanin through the action of the mirosynase enzyme, whose consumption has been directly related with a lower risk for neurodegenerative diseases, diabetes, atherosclerosis, and cancer [40].

#### 3.3.3. Isothiocyanates

**Figure 3:**
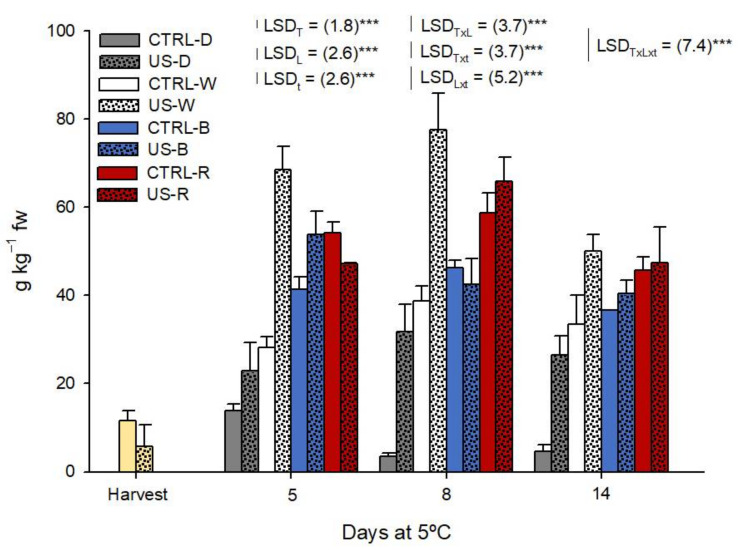
Sulforaphane content of rocket sprouts obtained from untreated (CTRL) and treated seeds (US) during a shelf life of 14 days at 5 °C under illumination with a photoperiod of 14 h fluorescent light + 10 h with several visible spectrum LEDs or in darkness. T: light treatment; t: sampling time; ***: *p* < 0.001.

Obtained results from sulforaphane analysis in the studied rocket sprouts are shown in Figure 3. As observed in the previous section, the glucoraphanin content in rocket sprouts represented 22% of the total content of these bio-compounds, from which sulforaphane derives. After assessing the remaining compounds, the photoperiod assayed was again effective in enhancing the production of these bioactive compounds, but what is remarkable in this case is that a remanent effect of the US seed pre-treatment is observed throughout the shelf life period under darkness and W photoperiods.

If we focus on the application of a US treatment before sowing the seeds, we can observe that when we used darkness in the photoperiod, the sulforaphane content of US sprouts increased ~4.7-fold compared to the CTRL sprouts. Furthermore, under W and B photoperiods, US-treated sprouts also reported increases by ~68% and ~58% in comparison with CTRL. Nevertheless, these positive effects were not appreciated when the rockets sprouts were stored under the R photoperiod.

Apart from that, the fact that is always worth repeating is the stimuli of the biosynthesis of phytochemicals under the different lighting photoperiods studied. Specifically on the batch of rocket sprouts obtained from CTRL seeds, we can observe as W, B, and R increased ~2.7-fold, ~3.6-fold, and even ~8-fold the content of sulforaphane compared to those sprouts stored under the darkness photoperiod, respectively. In a lower level, the US rocket sprouts reported increases of ~10% (W), ~28% (B), and ~29% (R) in comparison with darkness, which demonstrates again that for both these technologies, illumination with visible spectrum LEDs in a photoperiod and a US pre-treatment of seeds before sowing do not seem to share synergistic effects.

According to the results shown by Wang et al. [37] for broccoli seedlings, the sulforaphane content is also overstimulated by the effect of red light applied alone or combined with blue light. Both combined lights stimulated the glucoraphanin biosynthesis and the accumulation of sulforaphane because of the upregulation of MAM1, UGT74C1, and SOT18 [37]. Nevertheless, these authors reported that blue LEDs applied during the growth of broccoli seedlings were not able to increase the sulforaphane synthesis, which does not exclude the possibility that this light treatment could generate such an effect during the postharvest period of other brassica plants in their younger growth stages, as occurred in the present study.

Lastly, as confirmed by several authors, the consumption of vegetables rich in sulforaphane brings positive effects in the fight against cancer development [41,42]. For instance, the optima dose of broccoli can contribute to the intake of protective compounds such as sulforaphane, which can inhibit *Helicobacter pylori*-induced stomach cancer [43,44], pulmonary metastasis [45], prostate cancer [46], or reduce the risk of several kinds of cancer in humans [41,47]. Furthermore, the literature shows that 175 µmol/kg body weight [41] are needed to reach these beneficial effects. Hence, we can say that at least the daily consumption of ~15 g of rocket sprouts added to salads or fresh foods, included in a healthy, balanced diet, could help to prevent the development of these kind of chronic diseases. In this sense, we would recommend the consumption of rocket sprouts stored under a photoperiod with white, blue, or red LED lighting instead of turning off lights at nights, because their sulforaphane content is triple the normal amount found in these young plants under the usual refrigerated shelf-life conditions.

## 4. Conclusions

Our study showed that a continuous lighting postharvest photoperiod including visible spectrum LED lights, polychromatic (White), or monochromatic (Blue or Red), is able to increase the synthesis of secondary metabolites in rocket sprouts during shelf life. A postharvest photoperiod of 10 h under such illumination, instead of darkness, +14 h under a conventional fluorescent lighting, usually applied in supermarkets and food companies, showed important improvements in the content of all the monitored phytochemicals: antioxidants as phenolic acids and flavonoids, glucosinolates, and sulforaphane. Although US treatment induced beneficial effects on the sulforaphane content during shelf life, these results were not observed when combined with photoperiods under darkness or white LEDs, which rejects the synergistic behaviour between both technologies applied to rocket sprouts in the studied conditions: during pre-sowing or post-harvest periods.

## Figures and Tables

**Table 1:** Bioactive compounds quantification of the control (CTRL) and ultrasound (US)-treated rocket seeds.

	**Phenolics acids (mg g^−1^)**					
	**Gallic acid**	**Caffeic acid**	**Ferulic acid**		**Coumaric acid**	**Sinapic acid**
**CTRL**	3.56 ± 1.19	1.82 ± 0.15	1.81 ± 0.39		3.07 ± 0.85	2.97 ± 0.39
**US**	3.25 ± 1.12	0.45 ± 0.16	1.64 ± 0.12		2.15 ± 0.38	2.05 ± 0.69
	**Flavonoids (mg g^−1^)**					
	**Kaempferol** **derivative-1**	**Kaempferol** **derivative-2**	**Kaempferol derivative-3**	**Rutin**	**Quercetin**	**Quercetin** **derivative**
**CTRL**	2.02 ± 0.14	6.16 ± 0.51	4.15 ± 0.93	2.25 ± 0.43	11.28 ± 3.5	9.29 ± 2.01
**US**	1.88 ± 0.15	5.85 ± 0.19	3.59 ± 0.56	2.04 ± 0.27	2.31 ± 0.20	2.10 ± 0.22
	**Dsf-Glucosinolates (mg g^−1^)**					
	**Glucoraphanin**	**Sinigrin**	**Glucoerucin**	**4-Methoxy-Glucobrassicin**	**Glucobrassicin**	**Neoglucobrassicin**
**CTRL**	12.10 ± 0.12	-	23.54 ± 1.32	-	-	-
**US**	13.26 ± 0.11	-	25.66 ± 1.05	-	-	-
	**Sulforaphane (mg g^−1^)**					
**CTRL**	9.33 ± 2.10					
**US**	50.15 ± 4.89 *					

* Denotes significant differences *p* < 0.05.

**Table 2:** Phenolic acid (g kg**^−^**^1^ fw) changes in rocket sprouts from control (CTRL) and ultrasound-treated seeds (US) during a shelf life of 14 days at 5 °C under illumination with a photoperiod of 14 h fluorescent light + 10 h with several visible spectrum LEDs or in darkness.

Seed Treatment	Postharvest 10 h Illumination Photoperiod	Days at 5 °C	Gallic Acid	Caffeic Acid	Ferulic Acid	Coumaric Acid	Sinapic Acid	Total Phenolic Acids
**CTRL**	**-**	**Harvest**	0.73 ± 0.16	0.90 ± 0.15	0.58 ± 0.12	0.65 ± 0.14	0.27 ± 0.02	**3.14 ± 0.55**
**US**			0.39 ± 0.08	0.46 ± 0.16	0.29 ± 0.09	0.51 ± 0.16	0.15 ± 0.05	**1.80 ± 0.55**
**CTRL**	**Darkness**	**5**	0.41 ± 0.07	0.33 ± 0.03	0.20 ± 0.01	0.54 ± 0.03	0.15 ± 0.01	**1.63 ± 0.10**
		**8**	0.40 ± 0.07	0.30 ± 0.06	0.49 ± 0.09	0.68 ± 0.08	0.13 ± 0.01	**2.00 ± 0.29**
		**14**	0.36 ± 0.02	0.22 ± 0.01	0.36 ± 0.05	0.73 ± 0.07	0.14 ± 0.01	**1.80 ± 0.12**
	**White**	**5**	0.45 ± 0.07	0.44 ± 0.09	0.27 ± 0.06	0.65 ± 0.10	0.16 ± 0.01	**1.98 ± 0.32**
		**8**	0.52 ± 0.11	0.52 ± 0.06	0.59 ± 0.09	0.77 ± 0.06	0.15 ± 0.00	**2.56 ± 0.32**
		**14**	0.34 ± 0.03	0.34 ± 0.05	0.56 ± 0.13	1.03 ± 0.03	0.18 ± 0.01	**2.44 ± 0.19**
	**Blue**	**5**	0.45 ± 0.03	0.52 ± 0.05	0.32 ± 0.03	0.56 ± 0.06	0.15 ± 0.01	**2.00 ± 0.16**
		**8**	0.51 ± 0.09	0.56 ± 0.16	0.56 ± 0.14	0.79 ± 0.04	0.16 ± 0.02	**2.58 ± 0.09**
		**14**	0.35 ± 0.05	0.29 ± 0.04	0.83 ± 0.03	0.97 ± 0.06	0.15 ± 0.01	**2.59 ± 0.10**
	**Red**	**5**	0.42 ± 0.07	0.45 ± 0.10	0.33 ± 0.09	0.53 ± 0.07	0.14 ± 0.03	**1.87 ± 0.36**
		**8**	0.49 ± 0.05	0.59 ± 0.05	0.89 ± 0.18	0.87 ± 0.06	0.20 ± 0.01	**3.04 ± 0.23**
		**14**	0.54 ± 0.08	0.61 ± 0.02	1.15 ± 0.14	1.09 ± 0.10	0.21 ± 0.03	**3.59 ± 0.20**
**US**	**Darkness**	**5**	0.47 ± 0.01	0.45 ± 0.04	0.29 ± 0.04	0.51 ± 0.05	0.14 ± 0.01	**1.88 ± 0.07**
		**8**	0.44 ± 0.04	0.43 ± 0.03	0.59 ± 0.01	0.57 ± 0.05	0.20 ± 0.01	**2.23 ± 0.06**
		**14**	0.43 ± 0.05	0.38 ± 0.04	0.48 ± 0.02	0.67 ± 0.06	0.16 ± 0.01	**2.13 ± 0.14**
	**White**	**5**	0.48 ± 0.02	0.49 ± 0.07	0.26 ± 0.03	0.65 ± 0.11	0.15 ± 0.01	**2.03 ± 0.23**
		**8**	0.43 ± 0.05	0.37 ± 0.05	0.67 ± 0.14	0.82 ± 0.08	0.16 ± 0.02	**2.44 ± 0.27**
		**14**	0.51 ± 0.10	0.59 ± 0.12	1.06 ± 0.12	0.84 ± 0.15	0.21 ± 0.05	**3.21 ± 0.53**
	**Blue**	**5**	0.46 ± 0.03	0.48 ± 0.01	0.30 ± 0.02	0.59 ± 0.03	0.15 ± 0.01	**1.98 ± 0.02**
		**8**	0.39 ± 0.04	0.44 ± 0.01	0.56 ± 0.08	0.78 ± 0.09	0.16 ± 0.02	**2.34 ± 0.20**
		**14**	0.56 ± 0.15	0.63 ± 0.18	1.36 ± 0.16	1.09 ± 0.11	0.23 ± 0.01	**3.97 ± 0.30**
	**Red**	**5**	0.43 ± 0.02	0.51 ± 0.02	0.36 ± 0.01	0.57 ± 0.04	0.14 ± 0.01	**2.02 ± 0.06**
		**8**	0.60 ± 0.04	0.73 ± 0.06	0.53 ± 0.04	0.90 ± 0.04	0.18 ± 0.02	**2.89 ± 0.19**
		**14**	0.48 ± 0.04	0.54 ± 0.07	0.86 ± 0.12	0.86 ± 0.18	0.18 ± 0.02	**2.93 ± 0.13**

**Table 3:** Flavonoid (g kg**^−^**^1^ fw) changes of rocket sprouts from control (CTRL) and ultrasound-treated seeds (US) during a shelf life of 14 days at 5 °C under illumination with a photoperiod of 14 h fluorescent light + 10 h with several visible spectrum LEDs or in darkness.

Seed Treatment	Postharvest 10 h Illumination Photoperiod	Days at 5 °C	Kaempferol Derivative-1	Kaempferol Derivative-2	Kaempferol Derivative-3	Rutin	Quercetin	Quercetin Derivative	Total Flavonoids
**CTRL**	**-**	**Harvest**	0.31 ± 0.03	0.87 ± 0.09	0.66 ± 0.08	0.39 ± 0.03	1.90 ± 0.20	0.35 ± 0.04	**4.47 ± 0.48**
**US**			0.20 ± 0.06	0.59 ± 0.09	0.44 ± 0.13	0.29 ± 0.01	1.25 ± 0.38	0.21 ± 0.07	**2.97 ± 0.84**
**CTRL**	**Darkness**	**5**	0.19 ± 0.00	0.59 ± 0.04	0.45 ± 0.02	0.24 ± 0.01	1.46 ± 0.11	0.22 ± 0.00	**3.14 ± 0.18**
		**8**	0.18 ± 0.01	0.59 ± 0.09	0.41 ± 0.03	0.28 ± 0.04	1.58 ± 0.28	0.22 ± 0.02	**3.26 ± 0.48**
		**14**	0.17 ± 0.01	0.51 ± 0.04	0.36 ± 0.02	0.21 ± 0.03	1.33 ± 0.07	0.21 ± 0.01	**2.78 ± 0.17**
	**White**	**5**	0.22 ± 0.01	0.69 ± 0.09	0.54 ± 0.06	0.28 ± 0.02	1.70 ± 0.27	0.25 ± 0.02	**3.67 ± 0.47**
		**8**	0.20 ± 0.01	0.76 ± 0.03	0.48 ± 0.03	0.30 ± 0.02	1.98 ± 0.15	0.26 ± 0.01	**3.98 ± 0.23**
		**14**	0.21 ± 0.01	0.64 ± 0.05	0.44 ± 0.04	0.34 ± 0.05	1.71 ± 0.15	0.25 ± 0.01	**3.59 ± 0.29**
	**Blue**	**5**	0.19 ± 0.01	0.65 ± 0.06	0.52 ± 0.05	0.26 ± 0.02	1.67 ± 0.17	0.23 ± 0.02	**3.53 ± 0.32**
		**8**	0.21 ± 0.02	0.72 ± 0.05	0.46 ± 0.04	0.35 ± 0.02	1.96 ± 0.17	0.25 ± 0.02	**3.95 ± 0.30**
		**14**	0.18 ± 0.01	0.56 ± 0.02	0.38 ± 0.01	0.34 ± 0.03	1.54 ± 0.10	0.23 ± 0.00	**3.22 ± 0.16**
	**Red**	**5**	0.18 ± 0.03	0.60 ± 0.11	0.40 ± 0.12	0.26 ± 0.03	1.44 ± 0.13	0.21 ± 0.02	**3.10 ± 0.62**
		**8**	0.26 ± 0.01	0.92 ± 0.05	0.57 ± 0.02	0.46 ± 0.00	2.35 ± 0.09	0.35 ± 0.05	**4.89 ± 0.16**
		**14**	0.26 ± 0.02	0.84 ± 0.05	0.57 ± 0.05	0.51 ± 0.07	2.41 ± 0.09	0.33 ± 0.02	**4.91 ± 0.28**
**US**	**Darkness**	**5**	0.20 ± 0.01	0.64 ± 0.04	0.50 ± 0.04	0.24 ± 0.02	1.59 ± 0.15	0.22 ± 0.01	**3.39 ± 0.25**
		**8**	0.25 ± 0.01	0.74 ± 0.02	0.49 ± 0.01	0.34 ± 0.02	1.82 ± 0.08	0.29 ± 0.00	**3.94 ± 0.11**
		**14**	0.19 ± 0.01	0.60 ± 0.01	0.41 ± 0.01	0.30 ± 0.01	1.68 ± 0.04	0.24 ± 0.01	**3.40 ± 0.08**
	**White**	**5**	0.21 ± 0.02	0.68 ± 0.05	0.51 ± 0.01	0.28 ± 0.01	1.69 ± 0.14	0.24 ± 0.01	**3.61 ± 0.21**
		**8**	0.21 ± 0.01	0.72 ± 0.04	0.45 ± 0.05	0.38 ± 0.03	1.90 ± 0.12	0.26 ± 0.06	**3.93 ± 0.25**
		**14**	0.25 ± 0.05	0.78 ± 0.14	0.48 ± 0.09	0.48 ± 0.08	2.12 ± 0.32	0.31 ± 0.01	**4.43 ± 0.74**
	**Blue**	**5**	0.19 ± 0.00	0.63 ± 0.06	0.44 ± 0.01	0.26 ± 0.01	1.59 ± 0.12	0.23 ± 0.01	**3.34 ± 0.20**
		**8**	0.22 ± 0.01	0.70 ± 0.04	0.44 ± 0.02	0.36 ± 0.02	1.78 ± 0.07	0.26 ± 0.01	**3.75 ± 0.18**
		**14**	0.29 ± 0.01	0.91 ± 0.05	0.60 ± 0.02	0.58 ± 0.01	2.48 ± 0.08	0.35 ± 0.01	**5.20 ± 0.18**
	**Red**	**5**	0.20 ± 0.01	0.66 ± 0.03	0.43 ± 0.02	0.28 ± 0.02	1.63 ± 0.09	0.23 ± 0.01	**3.42 ± 0.16**
		**8**	0.24 ± 0.01	0.87 ± 0.03	0.54 ± 0.02	0.38 ± 0.01	2.27 ± 0.05	0.29 ± 0.01	**4.58 ± 0.13**
		**14**	0.24 ± 0.02	0.78 ± 0.10	0.50 ± 0.07	0.52 ± 0.10	2.27 ± 0.22	0.31 ± 0.03	**4.62 ± 0.84**

**Table 4:** LSD values for seed treatments (T: CTRL or US), lighting postharvest photoperiod (L: Darkness, White, Blue, or Red), and storage time at 5 °C (t: 0, 5, 8, and 14 days), and their interactions for phenolic acids and flavonoids of rocket sprouts.

	T	L	t	T × L	T × t	L × t	T × L × t
***Phenolic acids***							
Gallic acid	(0.03) **	ns.	(0.05) ***	ns	(0.07) ***	ns	ns
Caffeic acid	(0.04) *	(0.06) ***	(0.06) ***	ns	(0.08) ***	(0.12) *	(0.16) **
Ferulic acid	ns	(0.06) ***	(0.06) ***	(0.08) ***	(0.08) ***	(0.11) ***	(0.16) ***
Coumaric acid	(0.04) **	(0.06) ***	(0.06) ***	ns	(0.08) *	(0.12) **	ns
Sinapic acid	(0.01) ***	ns	(0.01) ***	ns	(0.02) ***	ns	ns
Total	(0.17) *	(0.25) ***	(0.25) ***	ns	(0.35) ***	ns	n.s
***Flavonoids***							
Kaempferol derivative-1	(0.01) *	(0.02) *	(0.02) ***	ns	(0.02) ***	(0.03) *	ns
Kaempferol derivative-2	ns	(0.05) ***	(0.05) **	ns	(0.08) ***	(0.11) *	ns
Kaempferol derivative-3	(0.03) **	ns	(0.04) ***	ns	(0.06) ***	(0.08) **	ns
Rutin	ns	(0.02) ***	(0.02) ***	(0.03) **	(0.03) ***	(0.04) ***	(0.06) ***
Quercetin	ns	(0.13) ***	(0.13) ***	ns	(0.18) ***	(0.26) ***	(0.36) *
Quercetin derivative	(0.01) **	(0.02) **	(0.02) ***	ns	(0.03) ***	(0.04) **	ns
Total	(0.12) **	(0.17) ***	(0.17) ***	(0.24) *	(0.24) ***	(0.34) ***	(0.49) ***

*: *p* < 0.05; **: *p* < 0.005; ***: *p* < 0.001; ns: not significant.

## Data Availability

Data sharing is not applicable to this article as no new data were created or analyzed in this article.

## Supplementary Materials

The following supporting information can be downloaded at: https://www.mdpi.com/article/10.3390/antiox11081490/s1, Figure S1. Light spectra used during postharvest storage. A photoperiod of 14 h of Fluorescence light + 10 h Darkness was used as control.
