# Supplementary material for: Ultrasounds and a Postharvest Photoperiod to Enhance the Synthesis of Sulforaphane and Antioxidants in Rocket Sprouts
Source: Antioxidants (Basel). 2022 Jul 29;11(8):1490. doi: 10.3390/antiox11081490 (PMC9404791; doi:10.3390/antiox11081490)
Supplement: Supplementary file 1 [file antioxidants-11-01490-s001.zip › antioxidants-1836030-supplementary.pdf]

Supplementary Material

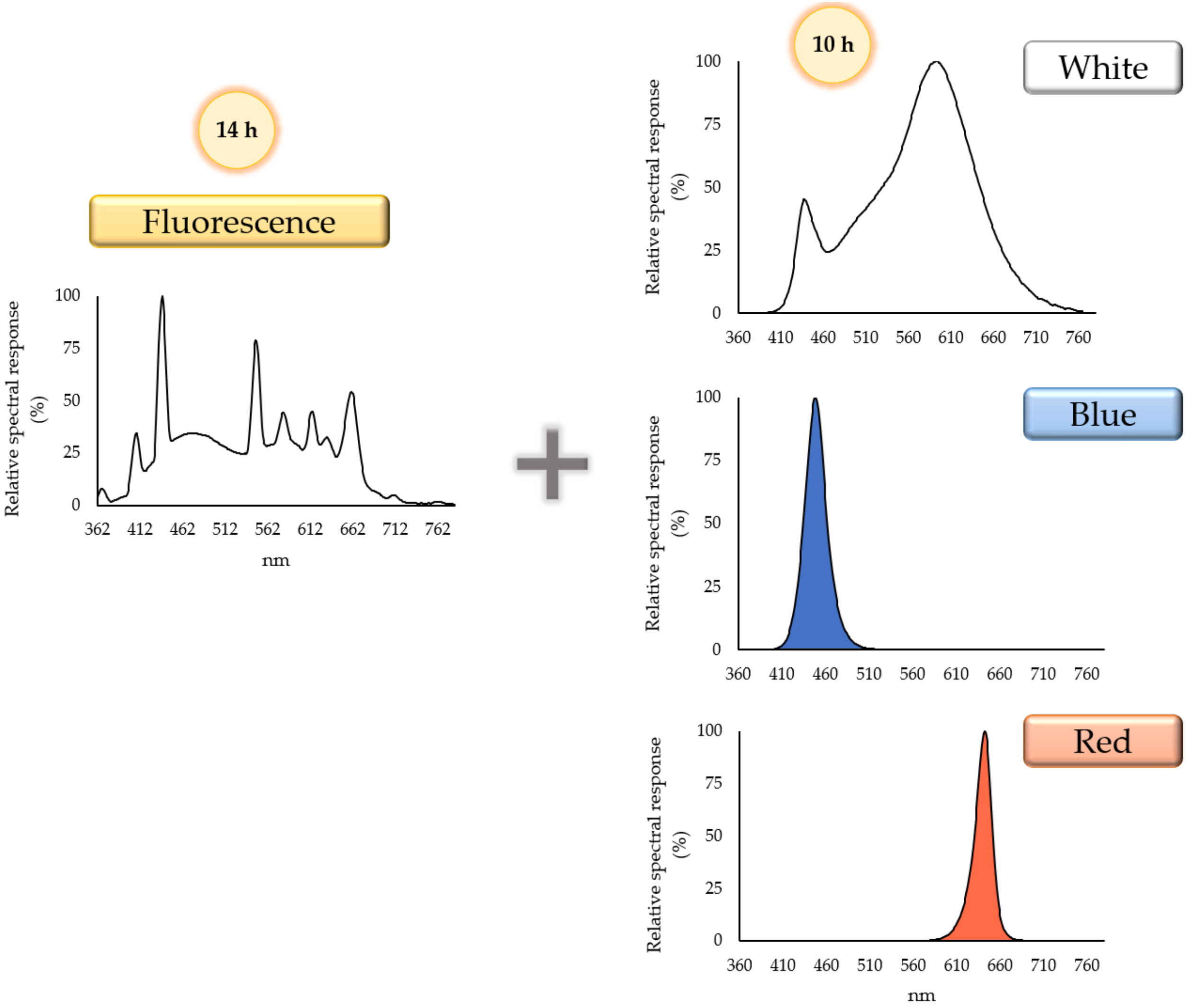

Figure S1. Light spectra used during postharvest storage. A photoperiod of 14 h Fluorescence light + 10 h Darkness was used as control.
